# Supplementary material for: Divalent metal cations stimulate skeleton interoception for new bone formation in mouse injury models
Source: Nat Commun. 2022 Jan 27;13:535. doi: 10.1038/s41467-022-28203-0 (PMC8795158; doi:10.1038/s41467-022-28203-0)
Supplement: Supplementary file 1 — Supplementary information [file 41467_2022_28203_MOESM1_ESM.pdf]

Supplementary Information

Divalent Metal Cations Stimulate Skeleton Interoception for New Bone Formation in Mouse Injury Models

Wei Qiao [1,2,3,4#], Dayu Pan [2,5#], Yufeng Zheng [6], Shuilin Wu [7], Xuanyong Liu [8,9], Zhuofan Chen [10], Mei Wan[2], Shiqin Feng [5], Kenneth M.C. Cheung [1,3], Kelvin W.K. Yeung [1,3*], Xu Cao [2*]

[1] Department of Orthopaedics and Traumatology, Li Ka Shing Faculty of Medicine, The University of Hong Kong, Hong Kong S.A.R., P.R. China

[2] Department of Orthopaedic Surgery, The Johns Hopkins University School of Medicine, Baltimore, MD 21205, USA

[3] Shenzhen Key Laboratory for Innovative Technology in Orthopaedic Trauma, The University of Hong Kong-Shenzhen Hospital, Shenzhen 518053, P.R. China

[4] Applied Oral Sciences, Faculty of Dentistry, The University of Hong Kong, Hong Kong S.A.R., P.R. China

[5] Department of Orthopaedics, Tianjin Medical University General Hospital, 154 Anshan Road, Heping District, Tianjin, China, 300052

[6] State Key Laboratory for Turbulence and Complex System and Department of Materials Science and Engineering, College of Engineering, Peking University, Beijing 100871, P.R. China

[7] School of Materials Science & Engineering, Tianjin University, Tianjin 300072, P.R. China

[8] State Key Laboratory of High Performance Ceramics and Superfine Microstructure, Shanghai Institute of Ceramics, Chinese Academy of Sciences, Shanghai 200050, P.R. China

[9] Cixi Center of Biomaterials Surface Engineering, Ningbo 315300, China

[10] Zhujiang New Town Clinic, Hospital of Stomatology, Sun Yat-sen University, Guangzhou, 510000, P. R. China

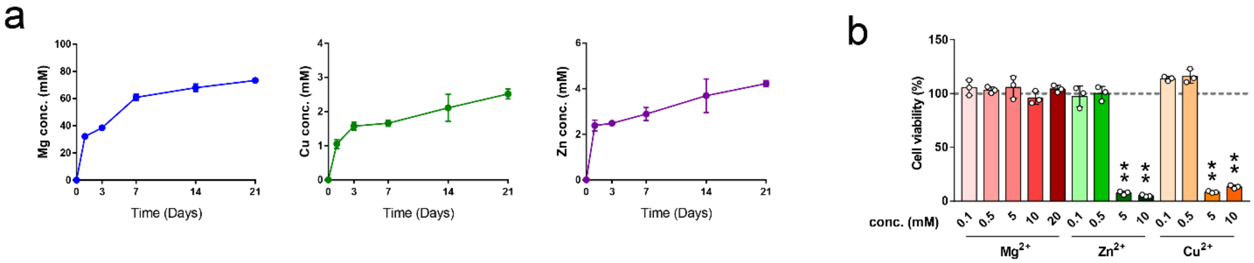

Supplementary Figure 1. (a) Cumulative divalent cation release from the cross-linked alginate measured *in vitro* using inductively coupled plasma mass spectrometry (n=3). **(b)** Cell viability of BMM cultured in medium supplemented with different concentrations of Mg^{2+}, Zn^{2+}, or Cu^{2+} (n=3). Data are mean ± s.d. **P*<0.05 or ***P*<0.01 by 1-way ANOVA with Tukey's post hoc test. Source data are provided as a source data file.

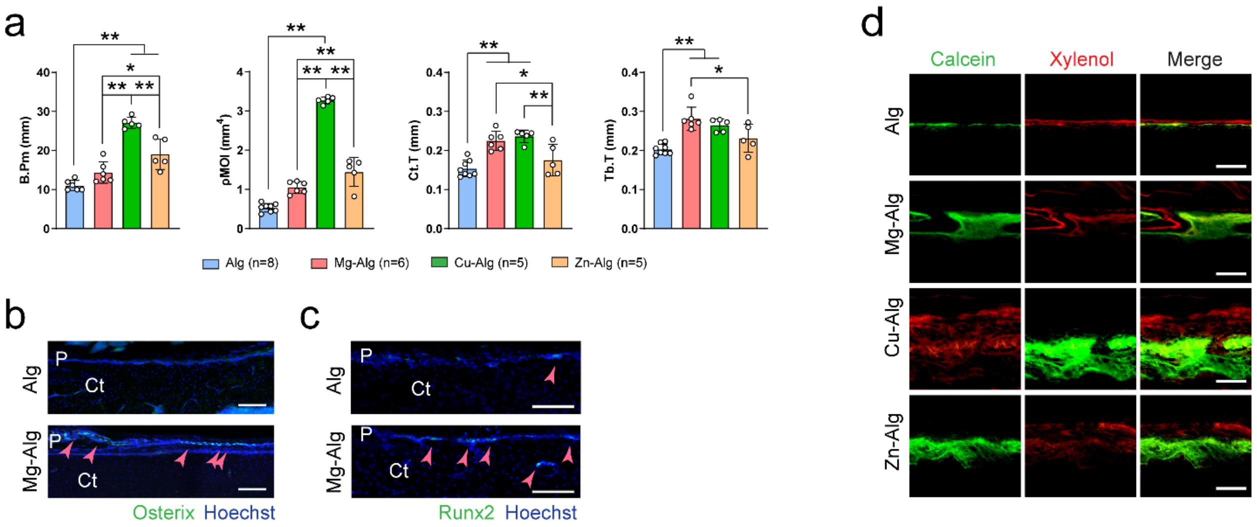

Supplementary Figure 2. (a) Quantitative measurements of bone perimeter (B.Pm), polar moment of inertia (ρMOI), cortical bone thickness (Ct.T), and trabecular thickness (Tb.T) of mouse femur grafted with pure alginate or divalent cation–releasing alginate. **(b, c)** Representative immunofluorescent images showing the presence of osterix$^+$ **(b,** n=3**)** or runt-related transcription factor 2$^+$ **(c,** n=3**)** osteoblasts on the cortical bone surface (scale bars = 100 μm). P, periosteum; Ct, cortical bone. **(d)** Representative images of calcein/xylenol labeling showing periosteal new bone formation (scale bars = 100 μm, n=3). Data are mean ± s.d. ***P*<0.01 by 1-way ANOVA with Tukey's post hoc test **(a)** or Student's T-test **(b)**. Source data are provided as a source data file.

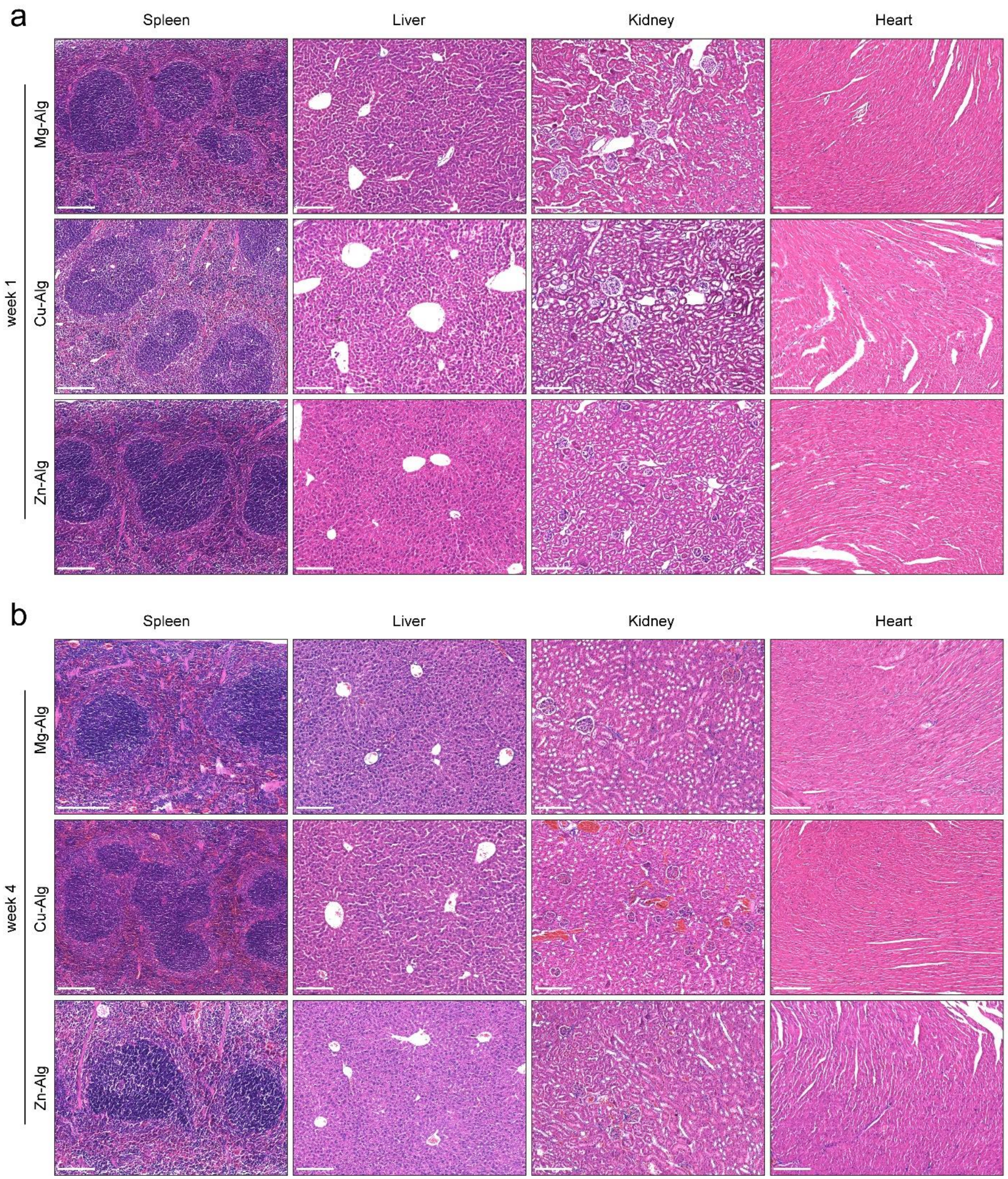

Supplementary Figure 3. (a, b) Representative H&E staining images showing the spleen, liver, kidney, and heart tissue at week 1 **(a,** n=3**)** and week 4 **(b,** n=4**)** after the operation (scale bars = 100 µm).

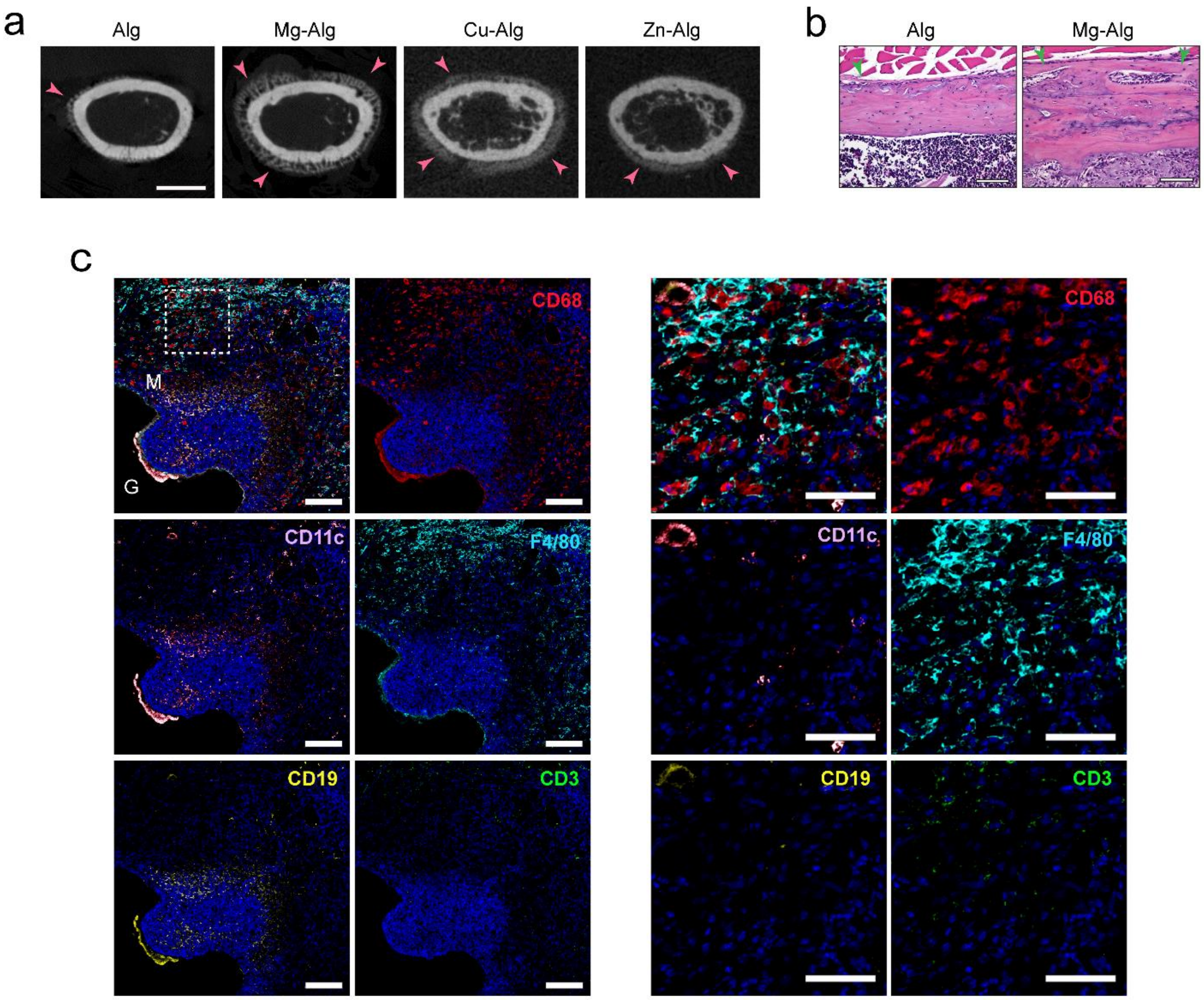

Supplementary Figure 4. (a) Reconstructed µCT images (scale bars = 1 mm) showing the cross-section of mouse femurs grafted with pure alginate (served as a control), Mg^{2+}-releasing alginate, Cu^{2+}-releasing alginate or Zn^{2+}-releasing alginate 7 days postoperatively. **(b)** Representative H&E staining images showing the newly formed woven bones on day 7 postoperatively (scale bars = 100 µm, n=3). **(c)** Representative multi-color immunofluorescent images showing the presence of immune cells around the divalent cation-releasing alginate (n=4). Images on the right side (scale bars = 50 µm) are high-resolution versions of the boxed regions in images on the left side (scale bars = 100 µm). M, bone marrow; G, grafted material.

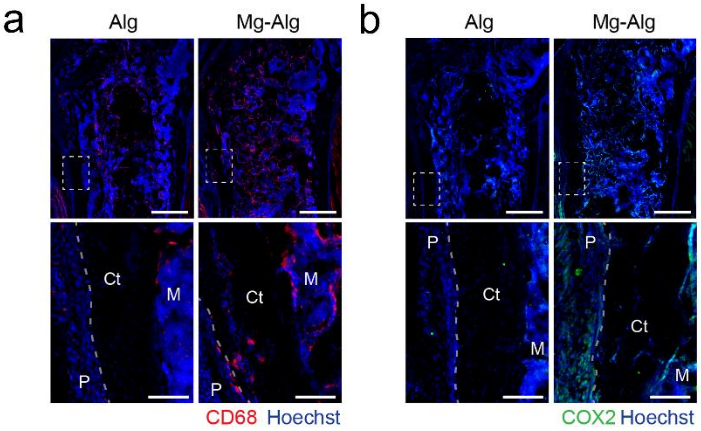

Supplementary Figure 5. (a, b) Representative immunofluorescent images showing the presence of $CD68^+$ macrophages **(a,** n=4**)** and the expression of COX2 **(b,** n=4**)** at week 1 postoperatively. Lower images (scale bars = 100 µm) are high-resolution versions of the boxed regions in the upper images (scale bars = 500 µm). P, periosteum; Ct, cortical bone; M, bone marrow.

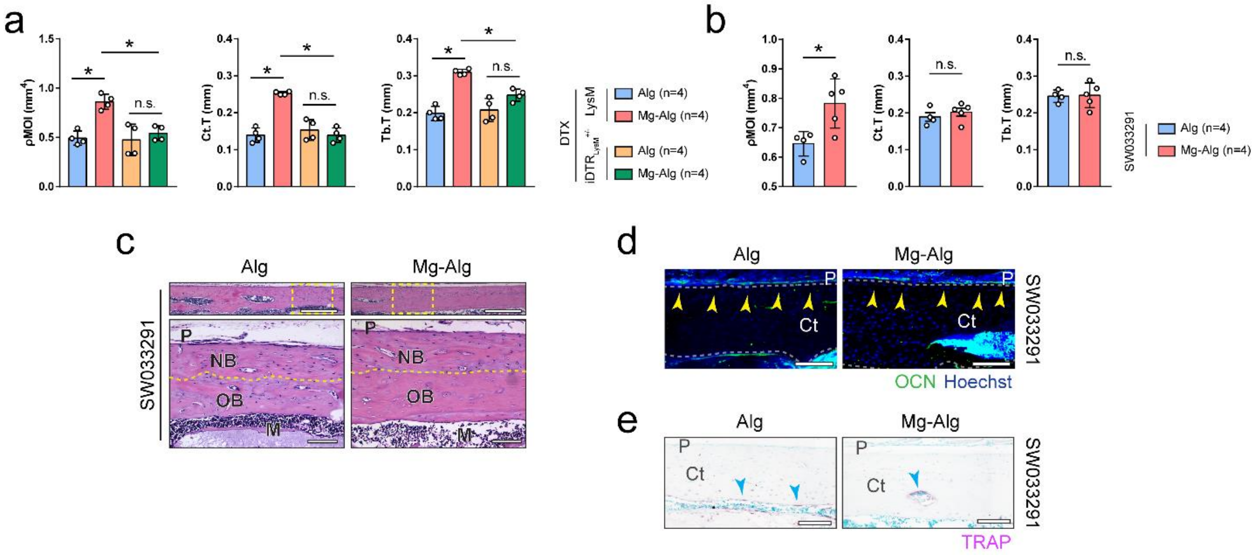

Supplementary Figure 6. **(a)** Quantitative measurements of ρMOI, Ct.T, and Tb.T of mouse femurs grafted with pure alginate or Mg^{2+}-releasing alginate after the administration of diphtheria toxin. **(b)** Quantitative measurements of ρMOI, Ct.T, and Tb.T of femurs from SW033291-injected mice. **(c)** Representative H&E staining images showing periosteal new bone formation in SW033291-injected mice (n=3). Lower images (scale bars = 100 μm) are high-resolution versions of the boxed regions in the upper images (scale bars = 500 μm). P, periosteum; NB, new bone; OB, old bone. **(d)** Representative immunofluorescent images showing the presence of OCN^{+} osteoblasts on the cortical bone surface (scale bars = 100 μm, n=3). **(e)** Representative TRAP staining images showing the presence of $TRAP^{+}$ osteoclasts on the cortical bone surface (scale bars = 200 μm, n=3) P, periosteum; Ct, cortical bone. Data are mean ± s.d. **P*<0.05 by 1-way ANOVA with Tukey's post hoc test **(a)** or Student's two-sided T-test **(b)**. Source data are provided as a source data file.

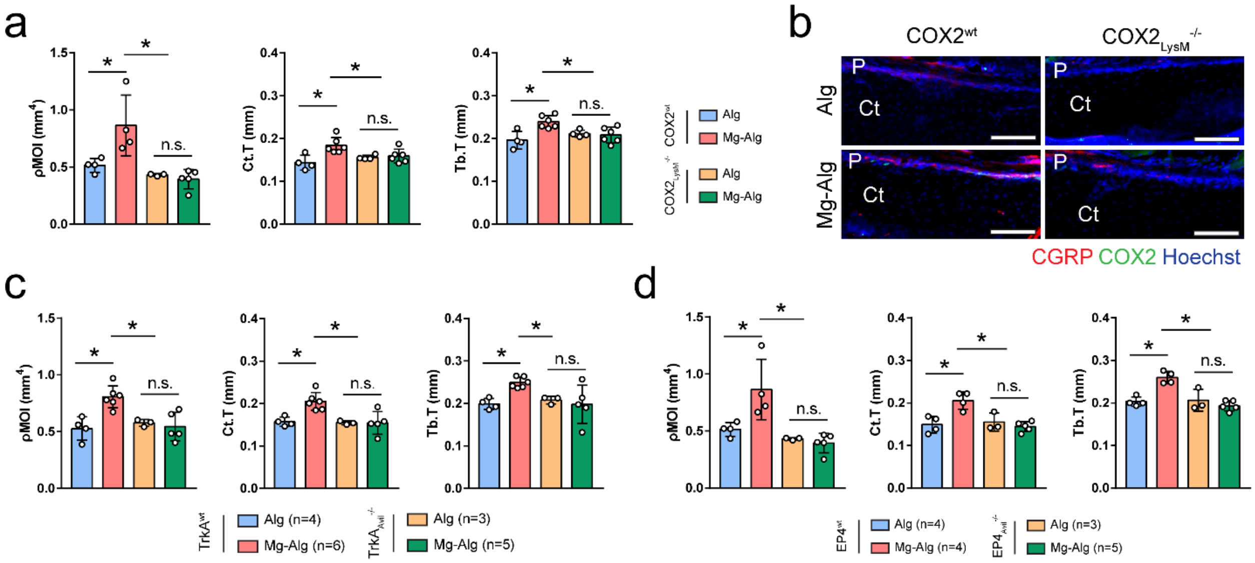

Supplementary Figure 7. (a) Quantitative measurements of ρMOI, Ct.T, and Tb.T of femurs from $COX2^{wt}$ or $COX2_{LysM}{}^{-/-}$ mice grafted with pure alginate or Mg^{2+}-releasing alginate. **(b)** Co-immunostaining of CGRP and COX2 in the periosteum of femurs from $COX2^{wt}$ or $COX2_{LysM}{}^{-/-}$ mice. (scale bars = 100 μm). P, periosteum; Ct, cortical bone. **(c)** Quantitative measurements of ρMOI, Ct.T, and Tb.T of femurs from $TrkA^{wt}$ mice or $TrkA_{Avil}{}^{-/-}$ mice grafted with pure alginate or Mg^{2+}-releasing alginate. **(d)** Quantitative measurements of ρMOI, Ct.T, and Tb.T of femurs from $EP4^{wt}$ or $EP4_{Avil}{}^{-/-}$ mice grafted with pure alginate or Mg^{2+}-releasing alginate. Data are mean ± s.d. *P<0.05 by 1-way ANOVA with Tukey's post hoc test. Source data are provided as a source data file.

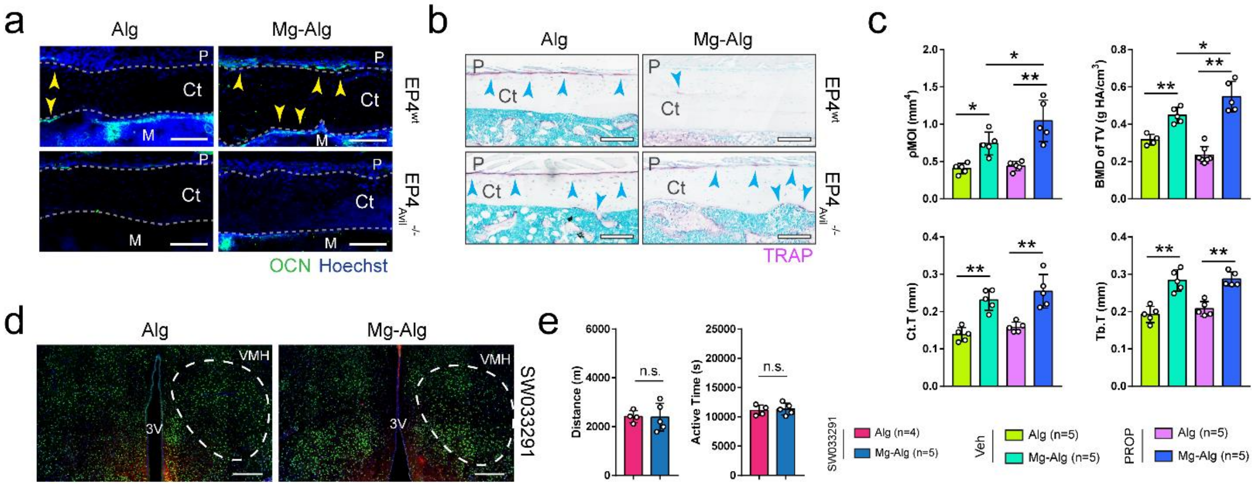

Supplementary Figure 8. (a) Representative immunofluorescent images showing the presence of OCN$^+$ osteoblasts on the cortical bone surface (scale bars = 100 μm). P, periosteum; Ct, cortical bone; M, bone marrow. **(b)** Representative TRAP staining images showing the presence of TRAP$^+$ osteoclasts on the cortical bone surface (scale bars = 100 μm). **(c)** Quantitative measurements of ρMOI, BMD of TV, Ct.T, and Tb.T of femurs from mice injected with vehicle or propranolol. **(d)** Representative immunofluorescent images showing the phosphorylation of CREB in the VMH of mice injected with SW033291 at week 1 postoperatively (scale bars = 200 μm). **(e)** The daily distance (m) and duration (s) of running-wheel activity of mice injected with SW033291 at week 4 postoperatively. Data are mean ± s.d. **P*<0.05, ***P*<0.01 by 1-way ANOVA with Tukey's post hoc test **(c)** or Student's two-sided T-test **(e)**. Source data are provided as a source data file.

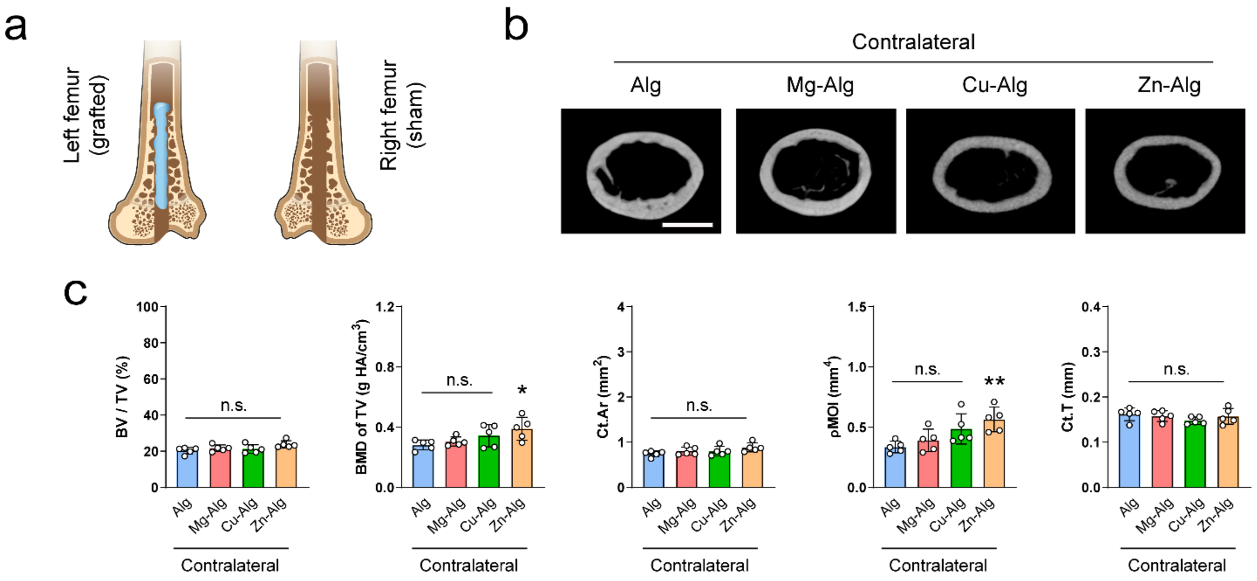

Supplementary Figure 9. (a) Divalent cation-releasing alginate was placed in the left femur and the contralateral femur was not grafted with anything after the surgical injury. **(b, c)** Reconstructed μCT images **(b,** scale bars = 1 mm**)** and corresponding measurements of BV/TV, BMD of TV, Ct.Ar, ρMOI, and Ct.T **(c)** showing the cross-section of the contralateral femur after the placement of divalent cation-releasing alginate in the left the femur. Data are mean ± s.d. **P*<0.05, ***P*<0.01, and n.s. (nonsignificant) by 1-way ANOVA with Tukey's post hoc test. Source data are provided as a source data file.

Supplementary Table 1. Primers used in the reverse transcription–quantitative polymerase chain reaction assays.

Gene	Primer	
PTGES	Forward:	5'-GGATGCGCTGAAACGTGGA-3'
	Reverse:	5'-CAGGAATGAGTACACGAAGCC-3'
COX2	Forward:	5'-TTCAACACACTCTATCACTGGC-3'
	Reverse:	5'-AGAAGCGTTTGCGGTACTCAT-3'
EP4	Forward:	5'-CGGTTCCGAGACAGCAAA-3'
	Reverse:	5'-CGGTTCGATCTAGGAATGG-3'
GAPDH	Forward:	5'-ATGTGTCCGTCGTGGATCTGA-3'
	Reverse:	5'-ATGCCTGCTTCACCACCTTCTT-3'

COX2, cyclooxygenase 2; EP4, prostaglandin E2 receptor 4; GAPDH, glyceraldehyde-3-phosphate dehydrogenase; PTGES, prostaglandin E Synthase
