# Supplementary material for: Divalent metal cations stimulate skeleton interoception for new bone formation in mouse injury models
Source: Nat Commun. 2022 Jan 27;13:535. doi: 10.1038/s41467-022-28203-0 (PMC8795158; doi:10.1038/s41467-022-28203-0)
Supplement: Supplementary file 3 — Reporting Summary [file 41467_2022_28203_MOESM3_ESM.pdf]

nature research

Corresponding author(s): Xu CAO

Last updated by author(s): Dec 20, 2021

Reporting Summary

Nature Research wishes to improve the reproducibility of the work that we publish. This form provides structure for consistency and transparency in reporting. For further information on Nature Research policies, see our Editorial Policies and the Editorial Policy Checklist.

Statistics

For all statistical analyses, confirm that the following items are present in the figure legend, table legend, main text, or Methods section.

n/a	Confirmed	
☐	☒	The exact sample size (*n*) for each experimental group/condition, given as a discrete number and unit of measurement
☐	☒	A statement on whether measurements were taken from distinct samples or whether the same sample was measured repeatedly
☐	☒	The statistical test(s) used AND whether they are one- or two-sided<br>*Only common tests should be described solely by name; describe more complex techniques in the Methods section.*
☐	☒	A description of all covariates tested
☐	☒	A description of any assumptions or corrections, such as tests of normality and adjustment for multiple comparisons
☐	☒	A full description of the statistical parameters including central tendency (e.g. means) or other basic estimates (e.g. regression coefficient) AND variation (e.g. standard deviation) or associated estimates of uncertainty (e.g. confidence intervals)
☐	☒	For null hypothesis testing, the test statistic (e.g. *F*, *t*, *r*) with confidence intervals, effect sizes, degrees of freedom and *P* value noted<br>*Give P values as exact values whenever suitable.*
☒	☐	For Bayesian analysis, information on the choice of priors and Markov chain Monte Carlo settings
☒	☐	For hierarchical and complex designs, identification of the appropriate level for tests and full reporting of outcomes
☒	☐	Estimates of effect sizes (e.g. Cohen's *d*, Pearson's *r*), indicating how they were calculated

Our web collection on statistics for biologists contains articles on many of the points above.

Software and code

Policy information about availability of computer code

Data collection	SkyScan (ver 1.0.12.0) was used for CT scan. Zeiss ZEN software (ver 3.4) was used for capture of immunofluorescence images.
Data analysis	NRecon (ver 1.7.0.4), CTAn (ver 1.16.9.0), CTvox (ver 3.2.0.0) were used for µCT analysis. GraphPad Prism 7, Microsoft Excel (Office 365), Adobe Photoshop CC, and Adobe Illustrator CC were used for performing general data analysis and generating figures. Zeiss ZEN software (ver 3.4) was used for analysis of immunofluorescent images. ImageJ (ver 1.50i) and were used for quantitative imaging analyses.

For manuscripts utilizing custom algorithms or software that are central to the research but not yet described in published literature, software must be made available to editors and reviewers. We strongly encourage code deposition in a community repository (e.g. GitHub). See the Nature Research guidelines for submitting code & software for further information.

Data

Policy information about availability of data

All manuscripts must include a data availability statement. This statement should provide the following information, where applicable:

- Accession codes, unique identifiers, or web links for publicly available datasets
- A list of figures that have associated raw data
- A description of any restrictions on data availability

All relevant data that support the findings of this study are available within the Article, Supplementary Information, Source Data file.

Field-specific reporting

Please select the one below that is the best fit for your research. If you are not sure, read the appropriate sections before making your selection.

- [x] Life sciences
- [ ] Behavioural & social sciences
- [ ] Ecological, evolutionary & environmental sciences

For a reference copy of the document with all sections, see nature.com/documents/nr-reporting-summary-flat.pdf

Life sciences study design

All studies must disclose on these points even when the disclosure is negative.

Sample size	This is an exploratory study with not enough prior data available for sample size calculations. Therefore, the sample size was generally estimated to detect an expected effect size >1.5 or higher with a power of 80%. This approach was sufficient to detect pronounced biological effects with high potential for translational value.
Data exclusions	The animals were excluded due to unexpected death (e.g. anesthesia) or euthanasia resulting from ethnic consideration (e.g. weight loss > 20%, pain score above threshold)
Replication	All experiments were performed with independent replicates as described in the figure legends
Randomization	Randomization was performed blindly among animals
Blinding	Investigators were blinded during the group allocation and data analysis for all experiments.

Reporting for specific materials, systems and methods

We require information from authors about some types of materials, experimental systems and methods used in many studies. Here, indicate whether each material, system or method listed is relevant to your study. If you are not sure if a list item applies to your research, read the appropriate section before selecting a response.

Materials & experimental systems

n/a	Involved in the study	
[ ]	[x]	Antibodies
[x]	[ ]	Eukaryotic cell lines
[x]	[ ]	Palaeontology and archaeology
[ ]	[x]	Animals and other organisms
[x]	[ ]	Human research participants
[x]	[ ]	Clinical data
[x]	[ ]	Dual use research of concern

Methods

n/a	Involved in the study	
[x]	[ ]	ChIP-seq
[x]	[ ]	Flow cytometry
[x]	[ ]	MRI-based neuroimaging

Antibodies

Antibodies used

Primary Antibodies for Histology
anti-OCN (ab93876, Abcam), anti-COX2 (ab15191, Abcam), anti-CD68 (ab125212, ab31630, Abcam), anti-CGRP (ab81887, Abcam), anti-PGP9.5 (ab108986), anti-SP7 (ab22552, Abcam), anti-RUNX2 (ab76956, abcam), anti-Tyrosine Hydroxylase (AB152, Merck Millipore), anti-Phospho-CREB (ab32096, abcam), anti-CREB (ab32515, abcam), anti-CD11c (#97585, CST), anti-F4/80 (#70076, CST), anti-CD19 (#90176, CST), anti-CD3 (#99940, CST)

Primary Antibodies for Western Blotting
anti- NF-κB p65 (#8241, CST), anti-Phospho-NF-κB p65 (#3033, CST), anti-Phospho-IκBα (#2859, CST), anti-IκBα (#4814, CST), anti-HTR2C (ab197776, abcam), mouse anti-β-actin (#8457, CST)

Secondary Antibodies for Histology
Alexa-Fluor 488-conjugated secondary antibody (A11008, ThermoFisher), Alexa-Fluor 647-conjugated secondary antibody (A21240, ThermoFisher)

Validation

Histology
anti-OCN (ab93876, Abcam) was validated for IHC staining of mouse sample. REF: Sun T et al. In situ bone regeneration with sequential delivery of aptamer and BMP2 from an ECM-based scaffold fabricated by cryogenic free-form extrusion. Bioact Mater 6:4163-4175 (2021).
anti-COX2 (ab15191, Abcam) was validated for IHC staining of mouse sample. REF: Sun Q et al. Parathyroid hormone attenuates osteoarthritis pain by remodeling subchondral bone in mice. Elife (2021).
anti-CD68 (ab125212, ab31630, Abcam) were validated for IHC staining of mouse sample. REF: Nürnberger S et al. Repopulation of

decellularised articular cartilage by laser-based matrix engraving. EBioMedicine 64:103196 (2021) & Irastorza-Lorenzo A et al. Evaluation of Marine Agarose Biomaterials for Tissue Engineering Applications. Int J Mol Sci 22:N/A (2021).
anti-CGRP (ab81887, Abcam) was validated for IHC staining of mouse sample. REF: Xie MX et al. ATF4 selectively regulates heat nociception and contributes to kinesin-mediated TRPM3 trafficking. Nat Commun 12:1401 (2021).
anti-PGP9.5 (ab108986) was validated for IHC staining of mouse sample. REF: Chen LH et al. Targeting interleukin-20 alleviates paclitaxel-induced peripheral neuropathy. Pain 161:1237-1254 (2020).
anti-SP7 (ab22552, Abcam)was validated for IHC staining of mouse sample. REF: Kegelman CD et al. YAP and TAZ Promote Periosteal Osteoblast Precursor Expansion and Differentiation for Fracture Repair. J Bone Miner Res 36:143-157 (2021).
anti-RUNX2 (ab76956, abcam) was validated for IHC staining of mouse sample. REF: Li B et al. Baicalein alleviates osteoarthritis by protecting subchondral bone, inhibiting angiogenesis and synovial proliferation. J Cell Mol Med 25:5283-5294 (2021).
anti-Tyrosine Hydroxylase (AB152, Merck Millipore) was validated for IHC staining of mouse sample. REF: Johnson V, Xiang M, Chen Z, Junge HJ. Neurite Mistargeting and Inverse Order of Intraretinal Vascular Plexus Formation Precede Subretinal Vascularization in Vldlr Mutant Mice. PLoS One. 2015 Jul 15;10(7):e0132013.
anti-Phospho-CREB (ab32096, abcam) was validated for IHC staining of mouse sample. REF: Chen, Hao, et al. "Prostaglandin E2 mediates sensory nerve regulation of bone homeostasis." Nature communications 10.1 (2019): 1-13.
anti-CREB (ab32515, abcam) was validated for IHC staining of mouse sample. REF: Chen, Hao, et al. "Prostaglandin E2 mediates sensory nerve regulation of bone homeostasis." Nature communications 10.1 (2019): 1-13.
anti-CD11c (#97585, CST) was validated for IHC staining of mouse sample. REF: Hassel, Chervin, et al. "Ductal Macrophages Predominate in the Immune Landscape of the Lactating Mammary Gland." Frontiers in Immunology 12 (2021).
anti-F4/80 (#70076, CST) was validated for IHC staining of mouse sample. REF: Kimishima, Yusuke, et al. "Clonal hematopoiesis with JAK2V617F promotes pulmonary hypertension with ALK1 upregulation in lung neutrophils." Nature communications 12.1 (2021): 1-18.
anti-CD19 (#90176, CST) was validated for IHC staining of mouse sample. REF: Guo, Shuyu, et al. "GATA4 as a novel regulator involved in the development of the neural crest and craniofacial skeleton via Barx1." Cell Death & Differentiation 25.11 (2018): 1996-2009.
anti-CD3 (#99940, CST) was validated for IHC staining of mouse sample. REF: Wang, Yu, et al. "Use of FVB Myc-CaP cells as an immune competent, androgen receptor positive, mouse model of prostate cancer bone metastasis." Journal of Bone Oncology 30 (2021): 100386.

Western Blotting
anti- NF-κB p65 (#8242, CST) was validated for Western Blotting of mouse sample. REF: Yue-Chun, Li, et al. "Vagus nerve plays a pivotal role in CD4+ T cell differentiation during CVB3-induced murine acute myocarditis." Virulence 12.1 (2021): 360-376.
anti-Phospho-NF-κB p65 (#3033, CST) was validated for Western Blotting of mouse sample. REF: Yue-Chun, Li, et al. "Vagus nerve plays a pivotal role in CD4+ T cell differentiation during CVB3-induced murine acute myocarditis." Virulence 12.1 (2021): 360-376.
anti-Phospho-IκBα (#2859, CST) was validated for Western Blotting of mouse sample. REF:"Vagus nerve plays a pivotal role in CD4+ T cell differentiation during CVB3-induced murine acute myocarditis." Virulence 12.1 (2021): 360-376.
anti-IκBα (#4814, CST) was validated for Western Blotting of mouse sample. REF: "Vagus nerve plays a pivotal role in CD4+ T cell differentiation during CVB3-induced murine acute myocarditis." Virulence 12.1 (2021): 360-376.
anti-HTR2C (ab197776, abcam) was validated for Western Blotting of mouse sample. REF: Chen H et al. Prostaglandin E2 mediates sensory nerve regulation of bone homeostasis. Nat Commun 10:181 (2019).
anti-β-actin (#8457, CST) was validated for Western Blotting of mouse sample. REF: Li, Ke, et al. "TRIB3 promotes MYC-associated lymphoma development through suppression of UBE3B-mediated MYC degradation." Nature communications 11.1 (2020): 1-20.

Animals and other organisms

Policy information about studies involving animals; ARRIVE guidelines recommended for reporting animal research

Laboratory animals

The Advillin-Cre (Avil-Cre) mouse strain was kindly provided by Xingzhong Dong (Department of Neuroscience, The Johns Hopkins University, Baltimore, MD, USA).
The TrkAfl/fl mouse strain was obtained from David D. Ginty (Department of Neurobiology, Harvard Medical School, Boston, MD, USA).
The LysM-Cre mice and iDTRfl/fl mouse strain was purchased from the Jackson Laboratory (Bar Harbor, ME, USA).
The COX2fl/fl mouse strain was provided by Harvey Herschman (Department of Biological Chemistry, University of California, Los Angeles, Los Angeles, CA, USA).
The EP4fl/ fl mouse strain was obtained from Brian L. Kelsall (Laboratory of Molecular Immunology, National Institutes of Health, Bethesda, MD, USA).
The Rosa26YFP reporter mouse strain was obtained from Centre for Comparative Medicine Research (CCMR), the University of Hong Kong.
Heterozygous male Avil-Cre mice were crossed with a female TrkAfl/fl or EP4fl/fl mouse. The offspring were intercrossed to generate the following genotypes: wild type (referred to as WT in the text), Avil-Cre (Cre recombinase expressed driven by Advillin advillin promoter), TrkAfl/fl (mice homozygous for TrkA flox allele, referred to as TrkAwt in the text), EP4fl/fl (mice homozygous for EP4 flox allele, referred to as EP4wt in the text), Avil-Cre::TrkAfl/fl (conditional deletion of TrkA receptor in a dvillin lineage cells, referred to as TrkAAvil−/− in the text), Avil-Cre::EP4fl/fl (conditional deletion of EP4 receptor in a dvillin lineage cells, referred to as EP4Avil−/− in the text).
Heterozygous male LysM-Cre mice were crossed with a female iDTRfl/fl mouse or a COX2fl/fl mouse. The offspring were intercrossed to generate the following genotypes: WT, LysM-Cre, iDTRfl/fl, COX2fl/fl mice (mice homozygous for COX2 flox allele, referred to as COX2wt in the text), LysM-Cre::iDTRfl/- (referred to as iDTRLysM+/− in the text), LysM-Cre::COX2fl/fl (conditional deletion of COX2 in monocyte-macrophage lineage, referred to as COX2LysM−/− in the text).
Homozygous male LysM-Cre mice were crossed with a female Rosa26YFP mouse to generate LysM-YFP mice.

Animals were housed in specificpathogen-free (SPF) conditions with free access to water and food,a 12 hour day-night cycle in the central animal facility of the Johns Hopkins University. The ambient temperature was 20 +/- 2 degrees Celsius and humidity was kept at 50 +/- 10 %.

Wild animals	no wild animals were used in the study
Field-collected samples	no field collected samples were used in the study
Ethics oversight	All animal experimental protocols and relevant ethical regulations were followed, and the study was approved by the Animal Care and Use Committee of The Johns Hopkins University, Baltimore, MD, USA.

Note that full information on the approval of the study protocol must also be provided in the manuscript.
